# Supplementary material for: Chromothripsis during telomere crisis is independent of NHEJ, and consistent with a replicative origin
Source: Genome Res. 2019 May;29(5):737–49. doi: 10.1101/gr.240705.118 (PMC6499312; doi:10.1101/gr.240705.118)
Supplement: Supplemental Material [file supp_gr.240705.118_Supplemental_file_1.zip › contigs/annotated_contigs/DB49/contig.2.DB49_length_676_mean_cov_12.9127218935.docx]

**DB49_length_676_mean_cov_12.9127218935**

```
CTTCAGCGTGAGGCAGGCTCGTTTGCCCCAGCTGTTTCTCTTAAAAGTTCTCCGATAAGTCATTGCCACCACTACTTCCAAATAGCGTA
 >chrY:59018293-59018588 - E=1e-135
TGTTAACTTTGAGTTAATTTTAGATAATCTCGGGTTAATTTAAACAATTTTGGCACCAACAACATGGTTAGGCTAGTCAGCTTGGATGG

GTATAGGAATCCGTCAATCTGGGTTGCTGTGTACCAGCTCTGGGACTGGGGGCAAGTTGTAGCAGCCCTCAGCCTCCTGCTTCACCTGT

ATCAGGGGAGGCTGAGACCCACCT|TGCA|CAGGTGAGTTAATGTCATGCTTCTCAAAGTATGGCCTGTGGAAGGAGCAGCCTGGCGCT
                         >chrY:59016859-59017203 + E=4e-174
CCCTTGCAGTATTTTTGACAGAAATGCAGACCACTGGGCCCCACCTCAGGAGTGTGGAGTTGTAGCCGGATCCCTTTCACTGGCACATT

AAAGTTAGAGCCACCCTAGGTTAATACACATCACTGACCGGCCTCAAGGAATAGGAGTGCTGGGTCTATCAGTCTCTCCCAACCATTCC

CTGGGCTTTGCCAAGTGCTTTCTGCCTGCCGAGCTCACTCTTGGATCAGATGGGGCCTCCCTGCCAGCAGTTCTACCTCATCCTCTCTG

CACCTAATTCAACT|TCTGAGGTCACATACGTCACGTCGGGTCTCGACCGCCTCTC
```
